# Analgesic potential of different available commercial brands of botulinum neurotoxin-A in formalin-induced orofacial pain in mice

**DOI:** 10.1016/j.toxcx.2021.100083

**Published:** 2021-09-02

**Authors:** Thays Crosara Abrahão Cunha, Ana Claudia Gontijo Couto, Eduardo Januzzi, Rafael Tardin Rosa Ferraz Gonçalves, Graziella Silva, Cassia Regina Silva

**Affiliations:** a Post-Graduated Program Genetics and Biochemistry, Institute of Biotechnology, Federal University of Uberlândia, Uberlândia, MG, Brazil; b Post-Graduated Program Orofacial Pain, CIODONTO, Belo Horizonte, MG, Brazil; c Orofacial Pain Department, MaterDei Hospital, Belo Horizonte, MG, Brazil

**Keywords:** Pain, BoNT-A treatment, Migraine, BOTOX, Orofacial pain

## Abstract

The use of botulinum neurotoxin-A (BoNT-A) is an alternative for the management of orofacial pain disorders. Although only Botox has labeled, there are other commercial brands available for use, among them: Dysport, Botulift, Prosigne, and Xeomin. The objective of the present study was to evaluate the possible differences in the antinociceptive effect evoked by different commercially available formulations of BoNT-A in an animal model of inflammatory orofacial pain induced by formalin injection. Male C57/BL6 mice (20–25 g) were submitted to the pre-treatment with five different commercial brands of BoNT-A (Botox, Botulift, Xeomin, Dysport, or Prosigne; with doses between 0.02 and 0.2 Units of Botulinum Toxin, in 20 μL of 0.9% saline) three days prior the 2% formalin injection. All injections were made subcutaneously into the right perinasal area. After formalin injections, nociceptive behaviors like rubbing the place of injection were quantified during the neurogenic (0–5 min) and inflammatory (15–30 min) phases. The treatment using Botox, Botulift, and Xeomin were able to induce antinociceptive effects in both phases of the formalin-induced pain animal model, however, Dysport and Prosigne reduced the response in neither of them. Our data suggest that the treatment using different formulations of BoNT-A is not similar in efficacy as analgesics when evaluated in formalin-induced orofacial pain in mice.

## Highlights

• Botulinum neurotoxin-a reduced formalin-induced orofacial pain in mice.
• There are differences in the analgesic potential of different available commercial brands of botulinum neurotoxin-A.
• Botox, Botulift, Xeomin demonstrated analgesic effect when evaluated in formalin-induced orofacial pain in mice.

## Graphical abstract

**Figure:**
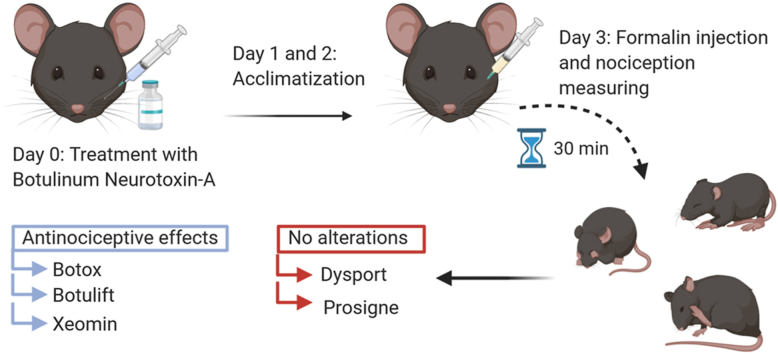

## Abbreviations:

BoNT-A: Botulinum Neurotoxin -A
SNAP-25: Synaptosomal-Associated Protein
VAMP: Vesicle-associated membrane protein 2
SNARE: Soluble N-ethylmaleimide Sensitive Factor Attachment Protein Receptor

## 1 Introduction

Pain is a common experience that has profound societal effects, with a greater prevalence in women that increases with age (Johannes et al., 2010). Orofacial pain (OFP) is extremely debilitating and refers to pain associated with the hard and soft tissues of the head, face, and neck (Groenewegen e Uylings, 2000), affecting about 26% of the population (Macfarlane et al., 2002). Anamnesis should be detailed, individualized, and comprehensive. Usually, the clinical approach is multidisciplinary, proportional to the degree and level of involvement and chronification, always starting from less to more invasive interventions. The treatment for OFP conditions is a significant issue and a challenge for the health care services and pharmaceutical industry. Ruling out the possibility of toothache, management of OFP consists of stabilizing plaque, pharmacotherapy, physiotherapy, in addition to thermotherapy, laser, needling, or anesthetic trigger point infiltration are recommended and efficient (Groenewegen e Uylings, 2000). However, some individuals are resistant and/or refractory to conventional approaches, motivating research in search of new therapeutic options, including the use of botulinum neurotoxin A (BoNT-A) (Chaurand et al., 2017; Schwartz e Freund, 2002; Scott et al., 2009; Sim, 2011).

Botulinum toxin is a neurotoxin produced by *Clostridium botulinum*, and there are seven (A – G) serotypes of botulinum toxin (Patil et al., 2016). The BoNT-A, initially intended to treat hyperactive movement disorders such as dystonia and blepharospasms, also demonstrated being effective to reduce dystonia-related pain and emerged as an alternative to treat chronic pain states as neuropathic pain, joint pain, back pain myofascial pain syndromes, migraine, and other headache types (Aoki, 2003; Dodick et al., 2010; Jankovic, 2018; Safarpour e Jabbari, 2018). The Food and Drug Administration approved the use of BoNT-A and BoNT-B for treatment in a variety of clinical and cosmetical conditions (Brin et al., 1989; Carruthers et al., 2002). In 2011, through the PREMPT protocol, the BoNT-A onabotulinum toxin A from Botox® (Allergan, Irvine, CA, USA), has been approved as a treatment for chronic migraine (Dodick et al., 2010). However, besides Botox®, at present, there are other BoNT-A products available worldwide as Botulift® (Medytox, Cheongwon, South Korea), Xeomin® (Merz Pharmaceuticals, Frankfurt, Germany), Dysport® (Ipsen, Paris, France), and Prosigne® (Lanzou Institute of Biological Products, Gansu, China), and their application for orofacial pain relieve has not yet been described.

Despite differences among these formulations, the therapeutic effects are believed to be addressed to the presence of the 150 kDa BoNT-A neurotoxin, released by the *C. botulinum* as a large complex with 900 kDa, consisting in accessory proteins, which are non-toxic non-hemagglutinin plus three hemagglutinins proteins, and a 150 kDa neurotoxin (Aoki et al., 2006; Dressler et al., 2018). This neurotoxin has a heavy (100 kDa) and light (50 kDa) chain. In the site of injection, the heavy chain (100 kDa) of BoNT-A seems to bind to acceptors consisting of gangliosides and synaptic vesicle 2 (SV2A-C) protein isoform and enters the neuron (Muraro et al., 2009). At the cytosol, the light chain (50 kDa) cleaves nine amino acids from SNAP-25 forming SNAP-25 (1–197). SNAP-25 (1–197) reacts with syntaxin, and VAMP-2/synaptobrevin and forms a SNARE complex, which competes with the normal SNARE complex, blocking the neurotransmitter release at the vesicular site (Matak et al., 2019; Pirazzini et al., 2017). For pain relief, some mechanisms have been proposed like the blockage of neurotransmitters release as acetylcholine (Pirazzini et al., 2017), glutamate (Da Silva et al., 2014), substance P (Chien et al., 2012; Purkiss et al., 2000), Calcitonin Gene-Related Peptide (CGRP) (Coelho et al., 2014; Lee et al., 2011), serotonin (Ibragić et al., 2016), gamma-aminobutyric acid (GABA) and enkephalin (McMahon et al., 1992), noradrenaline and dopamine (Ashton e Dolly, 1988), and glycine (Bigalke et al., 1981), all of them regulated by different targets as the activation of nociceptive receptors or SNARE modulation, for example.

Each of these BoNT-A formulations has a unique manufacturing process and contains different excipients (Pickett, 2014), and may be effective to treat different disorders (Baker e Nolan, 2017; Von Lindern et al., 2003). Thus, this study aimed to evaluate different BoNT-A commercially available formulations as possible analgesics in the treatment of an orofacial animal model of pain in mice.

## 2 Materials and methods

### 2.1 Animals

A total of 92 male C57/BL6 mice (20–25 g) were used in the experiments and were obtained from the Rodent Vivarium Network (REBIR) of the Federal University of Uberlândia (UFU). The animals were housed in cages at 22 °C with a 12:12 h light/dark cycle, with free access to food and water, and environmental enrichment. The animals were used only once. All the animals were euthanized using xylazine and ketamine, followed by cervical dislocation.

The experimental protocol was approved by the Ethics Committee in Animal Experimentation of the Federal University of Uberlândia (CEUA-UFU), approval number 92/2019, and performed in accordance with the National Institutes of Health guide for the care and use of Laboratory Animals (NIH Publications No. 8023, revised 1978). The number of animals is indicated for each group on the legend of the figure. To reduce the number of animals we used only 4 animals for the formalin control group in the majority of the experiments due to the reproducibility of the face rubbing in the animals*.* For more information see S1.

### 2.2 Drugs and reagents

For experiments, the following reagents and drugs were used: Botox® (Allergan, Irvine, CA, USA), Botulift® (Medytox, Cheongwon, South Korea), Xeomin® (Merz Pharmaceuticals, Frankfurt, Germany), Dysport® (Ipsen, Paris, France), Prosigne® (Lanzou Institute of Biological Products, Gansu, China), formalin (Sigma Aldrich, St. Louis, MO, USA), isoflurane (Cristália, São Paulo, Brazil) and saline.

### 2.3 Treatments

Right before treatment, the toxins were diluted in 0.9% saline (vehicle) as the manufacturer instructions described in the label. The animals were pre-treated with vehicle 20 μL (control group for pain development) or one of the five selected brands of Botulinum neurotoxin A, both injected subcutaneously at the right upper lip (perinasal area) using a 27 ½-gauge needle, 3 days prior to the formalin test. Before injections, the animals were firstly anesthetized with isoflurane 2%, supplemented with O_2_ 100%. Doses-response curves were constructed for Botox® (0.02, 0,06 e 0.2 Units of Botulinum Toxin in 20 μL of 0.9% saline), as described for Cui et al. (2004) and Luvisetto et al. (2006), with some modifications. For Dysport® (0.06, 0.18 e 0.6 Units of Botulinum Toxin in 20 μL of 0.9% saline) doses were selected based on the proportionality described on Dysport® leaflet and used in clinical management, following a 1:3 ratio (Botox®: Dysport®). Botulift®, Xeomin®, and Prosigne® were administered at 0,06 U/20 μL of saline, following the same proportionality in comparison to Botox® doses described in the clinic. Assuming that 100 units are equals to 5 ng of neurotoxin protein, our doses correspond to 1–10 pg/mouse (Cui et al., 2004). After the BoNT-A treatment, all animals were acclimatized to the experimental room for at least 1 h per day, during 3 days, preceding the formalin test. Each experiment was performed at least twice (duplicate), on different days, to reduce environmental risk, and with a different group of animals.

### 2.4 Formalin test

Orofacial nociception was induced by a subcutaneous injection of 20 μL of 2% formalin into the right perinasal area of animals briefly anesthetized with isoflurane (Luccarini et al., 2006). The nociception was quantified by measuring the time that the mouse spent rubbing the injected area with the paws for the first 5 min (considered the first phase, neurogenic), and 15–30 min (second phase, inflammatory) after the formalin injection. Results were expressed as the percentage of face rubbing, where formalin-inducing face rubbing was considered as being 100% of the response, to facilitate the comparison between the analgesic effects of the different brands.

### 2.5 Data analysis

All experiments submitted to this project had their sample size calculated using the G* Power 3.0.10 Software (0.25 effect size, error probability of 0.05, and proof power of 0.85). Kolmogorov-Smirnov normality test was used to determine whether the data values had normal distributions. For analysis, the data obtained with Botox® and Dysport® treatment were evaluated by a One-way analysis of Variance (ANOVA) followed by Dunnett post hoc test. The data obtained with the other treatments were evaluated by *t*-test. Were considered significant the differences with a P ≤ 0,05. All graphs and statistical analyses were performed using GraphPad Prism 5.0 (GraphPad Software, San Diego, CA).

## 3 Results

All animals submitted to the procedure were included in the statistical analysis. Only one mouse did not present the appropriate weight on the day of the experiment and was not submitted to intervention. Firstly, we verified if Botox® was able to prevent nociception in the orofacial pain animal model induced by formalin, which is regarded as being pertinent to clinical pain (Raboisson e Dallel, 2004), and which dose had the best therapeutic response. Based on this result, the dose to be tested for the other commercial brands was established as explained in the “treatment” subtitle, and the results are described below.

### 3.1 Antinociceptive effects of Botox® on formalin-induced orofacial pain

**Fig. 1:**
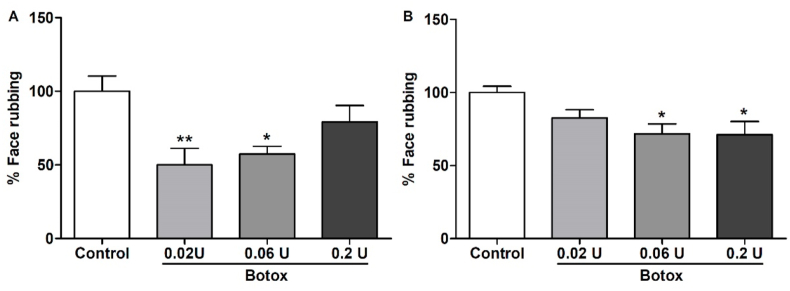
Effect of Botox® (0,02 U, 0,06 U, and 0,2U, n = 9 each group) on formalin-induced orofacial pain in mice (control n = 9. A) represents the first phase (neurogenic, 0–5 min) and B) represents the second phase (inflammatory, 15–30 min) of formalin-induced pain. Each column represents the mean ± S.E.M. One-way ANOVA followed by Dunnett's test. (*p < 0,05 and **p < 0,01 vs. Control group).

We observed that orofacial subcutaneous treatment using Botox® 0.02 U and 0.06 U reduced the face rubbing behavior by 42.9% and 34.5%, respectively; when analyzing the first phase of the formalin-induced orofacial pain (Fig. 1A). The second phase was prevented using Botox® at 0.06 U and 0.2 U, in 29.2% and 29.8%, respectively (Fig. 1B). Based on this experiment, we decided to use 0.06 U as the reference dose for the following experiments, when analyzing the possible analgesics effects of the other formulations of BoNT-A, performing the proportionalities of 1:3 and 1:1 with Botox, as described before.

### 3.2 Antinociceptive effects of Botulift® on formalin-induced orofacial pain

**Fig. 2:**
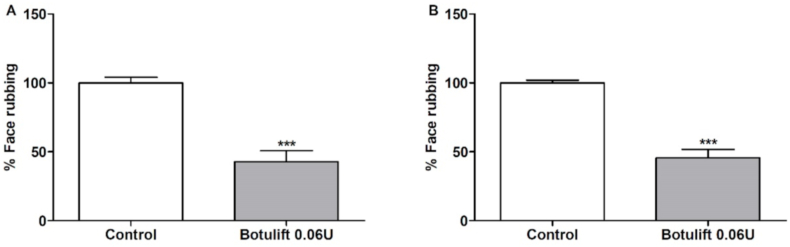
Effect of Botulift (Control, n = 4, Botulift, n = 8) on formalin-induced orofacial pain in mice. A) represents the first phase (neurogenic, 0–5 min) and B) represents the second phase (inflammatory, 15–30 min). Each column represents the mean ± S.E.M. Unpaired T-test. (***p < 0,001 vs Control group).

To evaluate Botulift® we used the same dose of Botox® (1:1) that has analgesic effects on both phases of the selected pain test. As we could observe, the treatment using Botulift® (0.06 U/20 μL) injection reduced the face-rubbing response at the first (57.2% of reduction) and second (54.5% of reduction) phase, compared to the control group (Fig. 2). Since our objective was not to make a comparison between BoNT-A analgesic potentials, but to evaluate if each formulation was able to elicit analgesic effects, we did not perform more doses of Botulift.

### 3.3 Antinociceptive effects of Xeomin® on formalin-induced orofacial pain

**Fig. 3:**
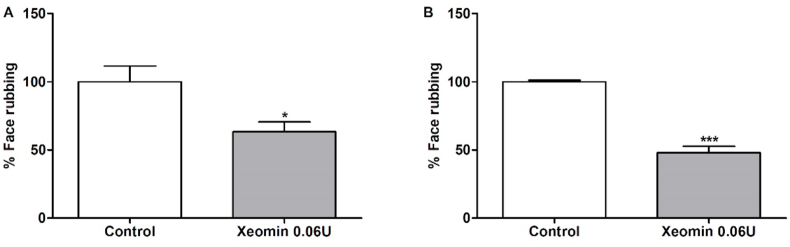
Effect of Xeomin® (Control, n = 4, Xeomin, n = 6) on formalin-induced orofacial pain in mice. A) represents the first phase (neurogenic, 0–5 min) and B) represents the second phase (inflammatory, 15–30 min). Each column represents the mean ± S.E.M. Unpaired T-test (*p < 0,05 and ***p < 0,001 vs. Control group).

The treatment using Xeomin® (0.06 U/20 μL) injection reduced the face-rubbing response at the first (37.5% of prevention) and second (51.9% of prevention) phase, compared to the control group (Fig. 3). Since our objective was not to make a comparison between BoNT-A analgesic potentials, but to evaluate if each formulation was able to elicit analgesic effects, we did not perform more doses of Xeomin®.

### 3.4 Antinociceptive effects of Dysport® on formalin-induced orofacial pain

**Fig. 4:**
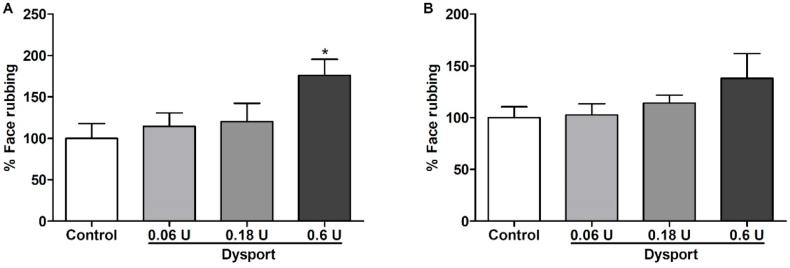
Effect of Dysport® (0,06 U, 0,18 U, and 0,6U, n = 6 per group) on formalin-induced orofacial pain in mice (control n = 6). A) represents the first phase (neurogenic, 0–5 min) and B) represents the second phase (inflammatory, 15–30 min) of formalin-induced pain. Each column represents the mean ± S.E.M. One-way ANOVA followed by Dunnett's test. (*p < 0.05 vs control group).

**Fig. 5:**
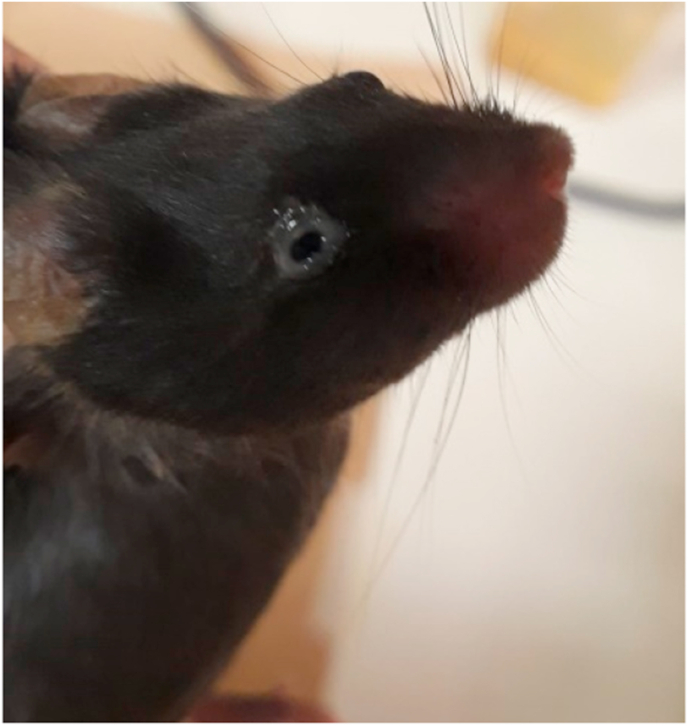
Adverse effect in the eye observed when animals were treated with Dysport 0,6U and Prosigne 0,06U.

Dysport® is used in the clinic at 1:3 in relationship to Botox® doses. As it was established that we would use the dose of 0.06U for Botox, based on references for mice treatment with BoNT-A, we adopted the concentration of 0.18U as a possible therapeutic dose for Dysport®. The treatment did not reduce the face-rubbing response of the first or second phase of formalin-induced orofacial pain when compared to the control group. The dose of 0.06U was then evaluated and no antinociceptive effects were observed. A third dose was tested, 0.6U, and it was observed an increase in face-rubbing response in the first (45,92%) and in the second phase (38%) in total rubbing time. (Fig. 4). We also observed adverse effects in the eyes of 3 of the 6 animals that used a dose of 0.6U, possibly related to the overdose of BoNT-A (Fig. 5).

### 3.5 Antinociceptive effects of Prosigne® on formalin-induced orofacial pain

**Fig. 6:**
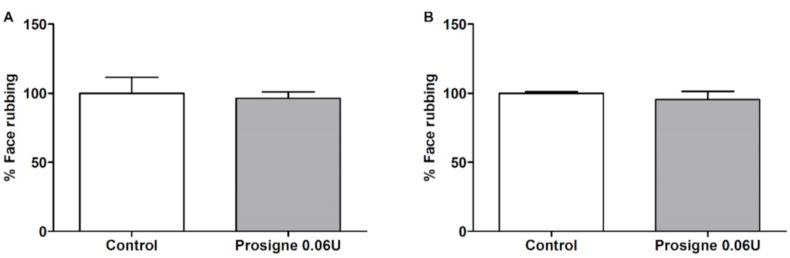
Effect of Prosigne® (Control, n = 4, Prossigne, n = 6) on formalin-induced orofacial pain in mice. A) represents the first phase (neurogenic, 0–5 min) and B) represents the second phase (inflammatory, 15–30 min). Each column represents the mean ± S.E.M. Unpaired T-test.

The treatment using Prosigne® (0,6 U/20 μL) injection was not able to prevent the development of face-rubbing response in the formalin orofacial pain test (Fig. 6) when compared to the control group. All the animals had an adverse effect on the eye, as shown in Fig. 4. Since our objective was not to make a comparison, but to evaluate if each formulation was able to elicit analgesic effects, we did not perform more doses of Prosigne®.

## 4 Discussion

This study evaluated the potential to induce antinociceptive effects for the following BoNT-A trademarks: Botox®, Botulift®, Xeomin®, Dysport®, and Prosigne®. Doses were based on previous animal studies using Botox for orofacial pain treatment and dilutions considered for humans treatments with BoNT-A. Our data suggest that the treatment using different formulations is not similar in efficacy as analgesics. Botox®, Botulift®, and Xeomin® were able to reduce the rubbing response in both, first and the second phase of formalin-inducing orofacial pain. Differently, Dysport® and Prosigne® did not reduce the pain response in any phase.

To study pain, different animal models are available and the formalin test is widely used as a preclinical pain model to investigate the analgesic effect of drugs (Dubuisson e Dennis, 1977). Usually accessed by subcutaneous intraplantar injections, it results in a series of behavioral responses, which are biphasic with an early short-lasting phase (phase 1), due to direct stimulation of nociceptors and inflammation start, followed by a second prolonged phase (phase 2) reflecting pain sensitization (Coderre et al., 1990; Dickenson e Sullivan, 1987). Formalin is also standardized as an animal model to study orofacial pain in rodents inducing face-rubbing episodes following the typical biphasic time-course seen in all formalin models (Clavelou et al., 1995; Luccarini et al., 2006). Since the orofacial region is one of the most densely innervated areas of the body, and these innervations are due to the trigeminal nerve, we used the animal model of formalin-induced orofacial pain for this study. Firstly, our experimental data support the analgesic effect of BoNT-A previously observed in an orofacial pain model induced by formalin in rodents (Magalhães et al., 2018; Matak et al., 2013; Silva et al., 2016). However, we observed analgesic effects for BoNT-A on the first phase of the formalin-induced orofacial pain, which was different from the previous findings demonstrated by Matak (2013). There are some differences between these studies that can reinforce these differences. Matak used rats and observed that animals spent rubbing around 40 s on the first phase of the formalin test, and 500 s on the second phase, while we observed this behavior for 100 and 200 s, respectively. Also, BoNT-A doses used in the studies were considerably different, 0,02–0,2 U for us and 1,25 U for Matak. Additionally, we evaluated the analgesic effects of BoNT-A 3 days after the toxin injection, and Matak observed these effects 6 days after treatments. Following, we could observe that the different formulations of BoNT-A exert different effects on the pain-behavioral responses induced by formalin.

The doses and protocol of treatment used here were based on the literature, where BoNT-A from Botox® shows analgesic effects when being administered in rodents three days before formalin injections and in these same doses (Cui et al., 2004; Luvisetto et al., 2006). Following, the doses for the different products of BoNT-A were defined using the conversion factor of 1:3 for Botox®: Dysport®, and 1:1 for Botox®: Botulift®, Prosigne®, and Xeomin®, as previously described (Barasnevicius Quagliato et al., 2010; Dressler et al., 2014; Odergren et al., 1998; Seo et al., 2015). Additionally, it is worth to be mention here that we observed some adverse effects in the animal eye when they were treated with the highest doses of Dysport® (0.6 U), and Prossigne® (0,06 U). The same adverse reactions in the eyes, as ptosis and dry eye, were previously described in the clinical trials done by the manufacturers, and this potential side effect is described at the label (Allergan Inc., 2011; IpsenBiopharm, 2009; MerzPharmaceuticals, 2010). Furthermore, we could observe that the analgesic effects of Botox in phase 1 decreased as we increase the concentration of Botox, probably due to adverse effects of high concentration of Botox, also indicated in humans where high doses of Botox are described as painful (Kazerooni e Armstrong, 2018). So, we do believe that the use of rodents to evaluate orofacial analgesic effects of BoNT-A is a strongly validated animal model to study pain.

Although our goal with this study was not to analyze the best BoNT-A for pain, instead to evaluate each one despite its possible effect as an analgesic for orofacial pain, some observations were made. Botulift® has demonstrated to have the best antinociceptive effect in formalin-inducing orofacial pain, reducing the face rubbing response in 57.2% at the first phase, and 54.5% at the second phase, in comparison with 37.5% (first phase) and 52% (second phase) of reduction for Xeomin® and 34,5% (first phase) and 29,2% (second phase) of reduction for Botox®. Certainly, these preclinical results need to be translated into clinical data, however, this study demonstrated, for the first time, possible orofacial analgesic effects being developed by other BoNT-A brands and with increased analgesia effects. On the other hand, we did not analyze the duration of the treatments, which is an important point to be observed in human analgesia.

This different potential as an analgesic is not surprising considering many factors related to BoNT-A manufacturing. The efficacy of different products can be affected by the diluent. Formulations using bulking agents and stabilizers, as gelatin phosphate buffer and human serum albumin, can be more potent. Some formulations can be more active and stable in saline than others (Brin et al., 2014; McLellan et al., 1996). Also, the potency of preparations of BoNT-A is calculated using an LD50 essay, and are expressed in mouse units, but there is no international reference standard against which potency is normalized, besides, each company uses a unique reference standard for the test (Dressler e Benecke, 2007; Hunt e Clarke, 2009). Because of the difference between the essays the units of biological activity of the commercial formulations of BoNT-A are not equivalent (Frevert et al., 2018; McLellan et al., 1996). Dressler (2011), using an LD50 essay compared the potency of five batches of Botox and Xeomin and observed that Botox has a potency of 103,1 MU (mouse units), and Xeomin has a potency of 101,7 MU (Dressler et al., 2012). The potency of Botox and Botulift were analyzed for Jeong et al. (2019), the estimated potency for Botox was 101,23% and for Botulift was 105,03% (Jeong et al., 2019).

Additionally, the difference in the manufacturing process originates products containing different amounts of the 150 KDa BoNT-A neurotoxin, which is described as responsible for mediating the therapeutic effects (Pickett, 2014; Pirazzini et al., 2017). The total amount of BoNT-A described in the literature, giving values of 5 ng per 100 U vial of Botox®, is related not only to the core of neurotoxin, but also the complexing proteins present in the formulation (Frevert, 2010; Panjwani et al., 2008). For example, Botox and Botulift have a 900 kDa complex, that is formed by botulinum neurotoxin (150 kDa), the non-toxic non-hemagglutinin, and three hemagglutinins proteins. Xeomin is formed only by the 150 kDa toxin. Dysport and Prosigne have a molecular mass ranging from 500 to 900 kDa. The presence of BoNT-A may impact the therapeutic effect: the lower amount of neurotoxin molecules, the lower cleave rate of SNAP-25. Field et al., in 2018 reported that Botox, Dysport, and Xeomin have different amounts of neurotoxin. In a 100 U vial of Botox there was 0,90 ng of BoNT-A, in a 100 U vial of Xeomin there was 0,4 ng and, in a 500 U vial of Dysport there was 2,69 ng of BoNT-A, resulting in 0.009 ng/U for Botox, 0.004 ng/U for Xeomin, and 0.005 ng/U for Dysport, respectively (Field et al., 2018). Additionally, the literature already demonstrated differences between the subtypes of BoNT-A (A1-8) related to their catalytic activity, toxicity, the capacity of entry neurons and elicit paralysis and there is a lack of information about the different BoNT-A subtypes present in these formulations (Christina L. Pier, Chen Chen, William H. Tepp, Guangyun Lin et al., 2011; Wang et al., 2013; Whitemarsh et al., 2013).

## 5 Conclusion

The analgesic treatment using different formulations of BoNT-A is not similar in efficacy. Here, the treatment using Botulift, Xeomin, and Botox had the analgesic response, while the treatment with Dysport and Prosigne did not show any antinociceptive effect. All the differences observed in the BoNT-A formulations, from the diluents used to the amount of neurotoxin, may affect the analgesic effects of the product. More preclinical and clinical studies comparing the effect of different preparations are required to elucidate the better treatment option for each case.

## Ethical statement

The authors declare that this work has not been published elsewhere. The animal experiments comply with the ARRIVE guideline and are carried out following the guideline for animal welfare.

## Declaration of competing interest

The authors declare that they have no known competing financial interests or personal relationships that could have appeared to influence the work reported in this paper.

## Credit author statement

Thays C Cunha: Conceptualization, Writing – original draft and supervision. Ana Claudia G. Couto: Investigation, Formal analysis, and writing- Original draft. Eduardo Januzzi: Conceptualization, Visualization and resources. Rafael T Gonçalves: Conceptualization, Visualization and resources. Graziella Silva: Conceptualization, Visualization and resources. Cássia Regina Silva: Project administration, Conceptualization, Methodology and writing - Review & Editing.
